# Fangchinoline induces G0/G1 arrest by modulating the expression of CDKN1A and CCND2 in K562 human chronic myelogenous leukemia cells

**DOI:** 10.3892/etm.2013.924

**Published:** 2013-01-24

**Authors:** YUPING WANG, JIE CHEN, LIN WANG, YUJI HUANG, YE LENG, GUIYING WANG

**Affiliations:** 1 Shanghai Key Laboratory of Signaling and Disease Research, School of Life Science and Technology, Tongji University, Shanghai 200092;; 2 Department of Biochemistry, School of Medicine, Jinggangshan University, Jiangxi 343009;; 3 Department of Hematology, Changhai Hospital, The Second Military Medical University, Shanghai 200433;; 4 Department of Endocrinology, East Hospital, Tongji University School of Medcine, Shanghai 200120, P.R. China

**Keywords:** fangchinoline, chronic myelogenous leukemia, G0/G1 arrest, cyclin-dependent kinase N1A, cyclin D2

## Abstract

Chronic myeloid leukemia (CML) is a hematopoietic stem cell disease caused by the oncoprotein BCR-ABL, which exhibits a constitutive tyrosine kinase activity. Imatinib mesylate (IM), an inhibitor of the tyrosine kinase activity of BCR-ABL, has been used as a first-line therapy for CML. However, IM is less effective in the accelerated phase and blastic phases of CML and certain patients develop IM resistance due to the mutation and amplification of the BCR-ABL gene. Fangchinoline, an important chemical constituent from the dried roots of *Stephaniae tetrandrae* S. Moore, exhibits significant antitumor activity in various types of cancers, including breast, prostate and hepatocellular carcinoma. However, the effects and the underlying mechanisms of fangchinoline in CML remain unclear. In the present study, we identified that fangchinoline inhibits cell proliferation in a dose- and time-dependent manner in K562 cells derived from the blast crisis of CML. Additional experiments revealed that fangchinoline induces cell cycle arrest at the G0/G1 phase and has no effect on apoptosis, which is mediated through the upregulation of cyclin-dependent kinase (CDK)-N1A and MCL-1 mRNA levels, as well as the downregulation of cyclin D2 (CCND2) mRNA levels. These findings suggest the potential of fangchinoline as an effective antitumor agent in CML.

## Introduction

Chronic myeloid leukemia (CML) is an acquired somatic mutation disorder of the hematopoietic stem cells (1) and is the third most common type of leukemia, constituting ∼15% of all leukemia cases. The natural course of CML is divided into three phases: chronic phase (CP), accelerated phase (AP) and blast phase (BP). CML typically begins with a CP. If left untreated, CML progresses from the CP to the AP after 3–5 years, followed by a terminal BP of lymphoid or myeloid phenotype (2). The only possible cure for CML is allogeneic stem cell transplantation. In the early CP, the 5-year survival rate following allogeneic transplantation is 25–70% (3); however, allogeneic stem cell transplantation is only available to a minority of individuals. Therefore, the main treatment for CML is chemotherapy and the conventional chemotherapeutic agents include imatinib mesylate (IM), interferon (IFN)-α, cytarabine and hydroxyurea. IFN with or without cytarabine was used as the conventional treatment for CP leukemia at the end of the 1990s. Currently, imatinib is the first-line therapy of choice for the CP of CML (4).

CML is characterized by the Philadelphia chromosome, which encodes the oncogene BCR-ABL. BCR-ABL is a fusion gene resulting from the reciprocal translocation between BCR and ABL on chromosomes 22 and 9, respectively (5,6). The chimeric protein BCR-ABL exhibits an uncontrolled tyrosine kinase activity and phosphorylates several substrates that activate multiple signaling pathways, including Ras, signal transducer and activator of transcription-5 (STAT-5), extracellular signal-regulated kinase (ERK)/mitogen-activated protein kinase (MAPK), Janus kinase 2 (JAK-2), phosphatidylinositol-3 kinase (PI-3K) and nuclear factor (NF)-κB (7,8). This abnormal signaling leads to the malignant cellular phenotype of CML, including increased proliferation, inhibition of the apoptotic response to mutagenic stimuli and reduction of adhesion to the bone marrow stroma and extracellular matrix (9). IM, an inhibitor of the tyrosine kinase activity of BCR-ABL, has been successfully used to treat CML patients in the CP and is considered the first-line therapy for the CP of CML (10–12). However, IM is less effective in the AP and BP of CML (13) and certain patients develop IM resistance (14) due to mutation and amplification of the BCR-ABL gene. Although inhibitors of farnesyltransferase and dual Src-family kinase/Abl kinase inhibit the growth of multi-drug resistant (MDR)-CML cells, side-effects and high cost limit their clinical application (15). Therefore, it is imperative to screen novel agents for the AP and BP of CML.

Fangchinoline is the main chemical constituents of *radix Stephaniae tetrandrae*, the dried roots of *Stephaniae tetrandrae* S. Moore (*Menispermaceae*), which has been shown to possess a wide range of pharmacological activities, including inhibition of histamine release and antihypertensive activities (16,17), anti-inflammatory effects (18–20), antiplatelet aggregation activities (21), antihyperglycemic actions (22,23), neuroprotective effects (24) and antioxidant and radical scavenging activities (25,26). Previous studies indicated that fangchinoline exhibited significant antitumor activity in various human cancers, including breast, prostate and hepatocellular carcinoma. Its antitumor mechanisms involved in inducing G1/S phase cell cycle arrest include inhibition of cyclin D1, upregulation of p27, potentiation of cancer cell apoptosis by upregulating pro-apoptotic B cell lymphoma (BCL)-2-associated X protein (BAX) and downregulating anti-apoptotic BCL-2, and initiation of excessive autophagy via p53/sestrin2/AMP-activated protein kinase (AMPK) signaling (27–29). Moreover, it has been reported that fangchinoline reverses the multidrug resistance of antitumor drugs mediated by P-glycoprotein (P-gp) (30,31). However, the effect of fangchinoline on CML and the underlying mechanisms remain unclear.

In the present study, we evaluated the effect of fangchino-line on the proliferation of K562 cells derived from the blast crisis of CML and investigated the potential mechanisms involved. We identified that fangchinoline efficiently inhibits the growth of K562 cells. Further investigation demonstrated that fangchinoline induces cell cycle arrest at G0/G1 rather than apoptosis, which may result from upregulation of cyclin-dependent kinase (CDK)-N1A and MCL-1, and down-regulation of cyclin D2 (CCND2). These findings suggest the possibility of fangchinoline as an effective antitumor agent in CML.

## Materials and methods

### Preparation of fangchinoline

Fangchinoline was kindly provided by Dr H.B. Wang (School of Life Science and Technology, Tongji University, Shanghai, China) and was stable when stored at 4°C. It was dissolved in dimethyl sulf-oxide (DMSO) as a stock solution and then stored at 4°C. The final DMSO concentration did not exceed 0.1% (v/v), which had no effect on cell growth in any experiment. Control cells were treated with the same amount of DMSO (0.1%, v/v) as used in the corresponding experiments.

### Cell culture

K562 cells purchased from Cell Bank Type Culture Collection of Chinese Academy of Sciences (Shanghai, China), were routinely maintained in RPMI-1640 culture medium (Thermo Fisher Scientific, Shanghai, China) supplemented with 10% fetal bovine serum (FBS; HyClone Laboratories Inc., Logan, UT, USA), 100 U/ml penicillin (Gibco BRL, Grand Island, NY, USA) and 100 μg/ml streptomycin (Gibco) and grown at 37°C in a humidified atmosphere of 5% CO_2_. The study was approved by the ethics committee of Tongji University, Shanghai, China.

### Cell proliferation assay

Cell proliferation was measured by direct counting. Initially, logarithmically growing cells were seeded into 6-well culture plates at a density of 2×10^5^ cells/ml and treated for 24 and 48 h at 0, 1, 3 and 10 *μ*M fangchinoline. The cells per well were collected and then counted using a hemocytometer.

### Cell viability assay

Cell viability was assessed using a methyl-thiazol tetrazolium (MTT) assay. Exponentially growing cells were inoculated into 96-well culture plates with 1×10^4^ cells per well and treated with a series of concentrations of fangchinoline (0, 0.1, 0.3, 1, 3 and 10 *μ*M) for 24 and 48 h. All experiments were conducted parallel with controls (0.1% DMSO). Then, 20 *μ*l sterile MTT (5 mg/ml, Sigma-Aldrich, St. Louis, MO, USA) was added to each well. Following further incubation at 37°C for 4 h, the reaction was stopped by adding 150 *μ*l DMSO. Following agitation on an automated shaker for 10 min, formazan production was determined by measurement of the spectrometric absorbance at 490 nm on an enzyme immunoassay analyzer FlexStation 3™ (Molecular Devices, Sunnyvale, CA, USA). The percentage of cell proliferation was calculated using the optical density (OD) as follows: (OD of experimental well - OD of blank well) / (OD of control well - OD of blank well) x100. The IC50 values, defined as the concentration of drug that caused 50% inhibition of absorbance compared with the control cell treated with DMSO only, were calculated using SPSS 17.0 statistical software (Aspire Software International, Leesburg, VA, USA).

### Cell cycle distribution analysis

Cell cycle distribution was determined by DNA staining with propidium iodide (PI). Exponentially-growing K562 cells were cultured and treated in 6-well culture plates (4×10^5^ cells/ml) with 0, 1, 3 and 10 *μ*M fangchinoline for 24 and 48 h. Cells were then harvested by centrifugation at 1,200 rpm at 4°C, washed once in phosphate-buffered saline (PBS) and fixed in 70% cold ethanol overnight. Cells were centrifuged and resuspended in 0.25 ml PBS containing 0.2 mg/ml RNase and incubated at 37°C for 1 h. Cells were added to 2.5 *μ*l 4 mg/ml PI and stored in the dark at 4°C. The cells were analyzed on a flow cytometer (Becton-Dickinson, San Jose, CA, USA) and the percentage of cells in the different phases of the cell cycle was analyzed using FlowJo software.

### Measurement of apoptosis by flow cytometry

Apoptotic cells were detected using an Annexin-V-Fluos staining kit. Cells were seeded in 6-well culture plates at a density of 4×10^5^ cells/ml, followed by fangchinoline and DMSO (control) treatment for 24 and 48 h. Following treatment, cells were collected and washed with PBS. After centrifugation at 200 x g for 5 min, the cell pellet was resuspended in 100 *μ*l Annexin-V-Fluos labeling solution (20 *μ*l Annexin-V-Fluos labeling reagent prediluted in 1 ml incubation buffer and 20 *μ*l PI), incubated for 15 min in the dark at room temperature and then immediately analyzed with a flow cytometer (Becton-Dickinson).

### RNA extraction and quantitative reverse transcription-polymerase chain reaction (RT-PCR)

Total RNA from each group of cells was extracted with TRNzol-A*^+^* reagent (Tiangen, Beijing, China). The first cDNA strand was synthesized using TIANScript RT Kit (Tiangen) and Oligo(dT) 15 primer from 2 *μ*g total RNA, according to the manufacturer’s instructions. The primer sequences for the target genes were as follows: CDKN1A, 5’-CTCATCCCGTGTTCTCCTTT-3′ (forward) and 5′-GTACCACCCAGCGGACAAGT-3′ (reverse); CCND2, 5′-TGGAGCTGCTGTGCCACG-3′ (forward) and 5′-GTGGCCACCATTCTGCGC-3′ (reverse); MCL-1, 5′-GGACATCAAAAACGAAGACG-3′ (forward) and 5′-GCAGCTTTCTTGGTTTATGG-3′ (reverse); BAX, 5′-GATGCGTCCACCAAGAAGCT-3′ (forward) and 5′-CGGCCCCAGTTGAAGTTG-3′ (reverse); β-actin, 5′-GGCTGTATTCCCCTCCATCG-3′ (forward) and 5′-CCAGTTGGTAACAATGCCATGT-3′ (reverse). The PCR amplifications were performed for 40 cycles of 95°C for 5 sec, 60°C for 20 sec and 72°C for 10 sec. Real-time quantitative RT-PCR was performed on a Stratagene Mx3000P system (Stratagene, La Jolla, CA, USA) with SYBR*^®^* Premix Ex Taq Mix (Takara Biotechnology Co., Ltd., Dalian, China). When cycling was completed, melting curve analysis was performed to establish the specificity of the PCR product. Data were collected and stored in Excel format and analyzed using Mx3000P software version 4.0. The expression level of cDNA of each candidate gene was internally normalized using β-actin. The relative quantitative value was expressed using the 2^−ΔΔCt^ method (32), representing the amount of candidate gene expression with the same calibrators. Each experiment was performed in duplicates and repeated three times.

### Statistical analysis

The data were analyzed by one-way analysis of variance using SPSS 17.0 software and P-values were calculated. Final values are expressed as mean ± standard deviation (SD). P<0.05 was considered to indicate a statistically significant difference.

## Results

### Fangchinoline inhibits K562 cell proliferation in a dose- and time-dependent manner

**Figure 1:**
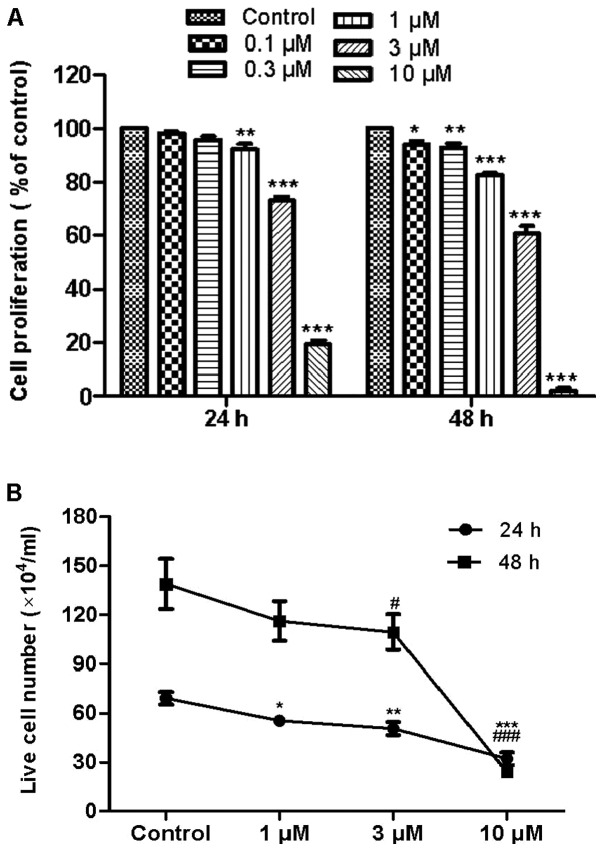
Fangchinoline inhibited cell proliferation in K562 Cells. (A) Cells were exposed to fangchinoline at various concentrations (0.1–10 *μ*M), incubated for 24 and 48 h and analyzed by methyl-thiazol tetrazolium (MTT) assay. (B) Cells were exposed to fangchinoline at various concentrations (1–10 *μ*M) and incubated for 24 and 48 h. The cells were collected and then counted using a hemocytometer. Data are presented as mean ± standard deviation (SD; n=3). ^*^ or ^#^ (P<0.05), ^**^ (P<0.01), and ^***^ or ^###^ (P<0.001) significant difference between the control and fangchinoline-treated groups.

To evaluate the effects of fangchino-line on CML cell proliferation, exponentially-growing K562 cells were treated with 0.1, 0.3, 1, 3 and 10 *μ*M fangchinoline. After 24 and 48 h, cell proliferation was determined by the MTT assay. Results revealed that fangchinoline significantly decreased the percentage of viable cells as compared with cells without treatment (Fig. 1A). After incubation with 1 *μ*M fangchinoline for 24 and 48 h, cell viability reduced to 92 and 83%, respectively. After incubation with 3 *μ*M fangchinoline for 24 and 48 h, cell viability reduced to 73 and 61%, respectively. After incubation with 10 *μ*M fangchino-line for 24 and 48 h, cell viability reduced to 19 and 2%, respectively. The IC50 of fangchinoline treatment for 24 and 48 h was ∼4.82 and 2.65 *μ*M, respectively. To verify the anti-proliferation effect of fangchinoline, cell proliferation was also measured by direct counting (Fig. 1B), which is consistent with the results of the MTT assay. Those results indicated that the anti-proliferation effect of fangchinoline on K562 cells is in a dose- and time-dependent manner.

### Fangchinoline induces cell cycle arrest at the G0/G1 phase in K562 cells

**Figure 2:**
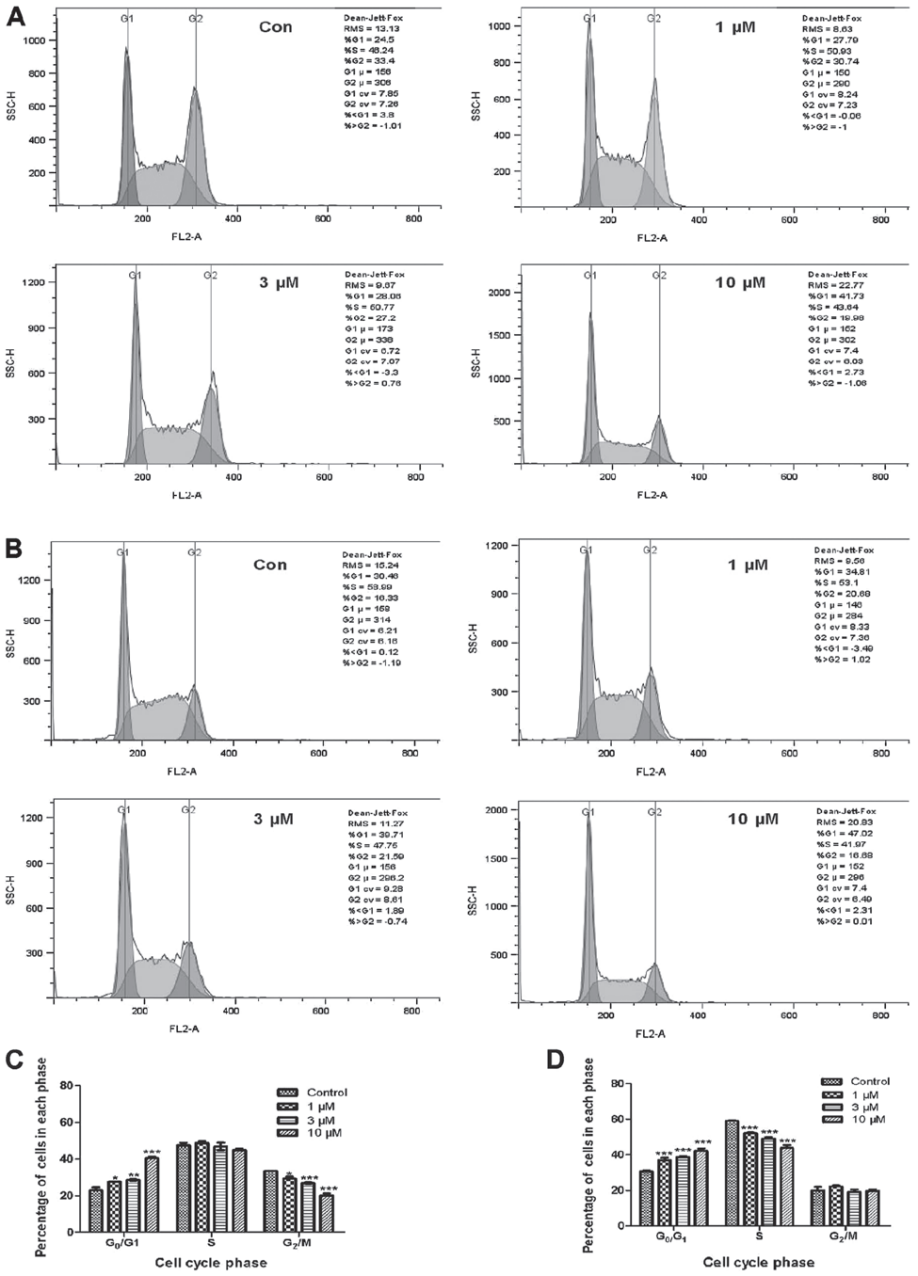
Fangchinoline induced cell cycle arrest in K562 Cells. (A) Cells were treated with and without the indicated concentrations of fangchinoline for 24 h. Then, the treated cells were harvested, stained with propidium iodide (PI) and examined by fluorescence-activated cell sorting (FACS). The percentages of cells in each phase of the cell cycle are shown. (B) Cells were treated with and without the indicated concentrations of fangchinoline for 48 h. (C) Cells were treated with and without the indicated concentrations of fangchinoline for 24 h. (D) Cells were treated with and without the indicated concentrations of fangchinoline for 48 h. Results of FACS are expressed as percentages of cells in each phase ± standard deviation (SD) for three independent experiments.

We subsequently analyzed cell cycle distribution in fangchinoline-exposed cells using flow cytometry following PI staining. Treatment with fangchinoline for 24 and 48 h resulted in the accumulation of K562 cells in the G0/G1 phase and a concomitant depletion of cells in the G2/M or S phases (Fig. 2A–D). After treatment with 1, 3 and 10 *μ*M fangchinoline for 24 h, the proportion of cells in the G0/G1 phase increased gradually from 23.0±2.05% in the control group to 27.6±0.29, 28.6±0.86 and 40.6±1.06%, respectively. The proportion of cells in the G2/M phase was reduced by ∼4, 7 and 13%, respectively, as compared to the control value (27.1±0.8%; Fig. 2A and C). After treatment with 1, 3 and 10 *μ*M fangchinoline for 48 h, the proportion of cells in the G0/G1 phase increased gradually from 30.7±0.96 to 37.1±2.1, 38.7±0.89 and 42.3±2.3%, respectively. The proportion of cells in the S phase was reduced by ∼7, 10 and 15%, respectively, as compared to the control value (59.0±0.31%; Fig. 2B and D). These results indicate that fangchinoline induces cell cycle arrest at the G0/G1 phase in K562 cells.

### Effects of fangchinoline on the expression level of cell cycle-related genes

**Figure 3:**
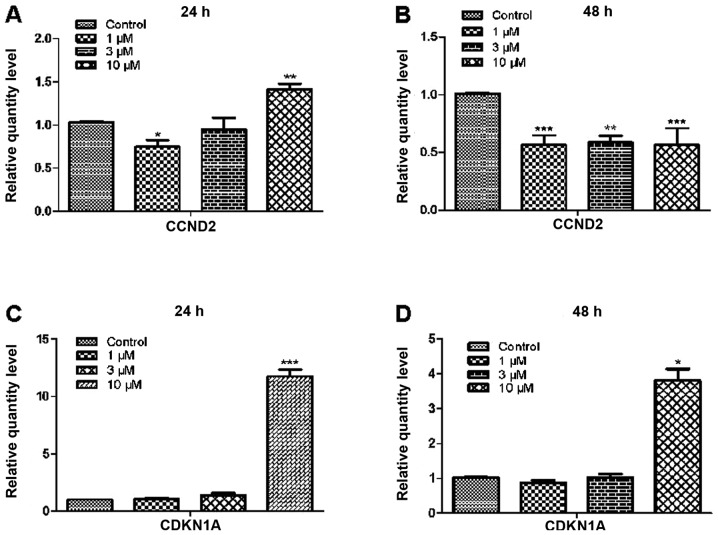
Effect of fangchinoline on cell cycle regulation-related gene expression. After treatment with and without the indicated concentrations of fangchinoline for 24 and 48 h, the mRNA levels of cell cycle regulation-related genes in each group of cells were examined by quantitative reverse transcription-polymerase chain reaction (RT-PCR), with the β-actin gene as the internal control. Data are presented as mean ± standard deviation (SD) for three-independent experiments. (A) The relative mRNA level of cyclin D2 (CCND2) after treatment for 24 h. (B) The relative mRNA level of CCND2 after treatment for 48 h. (C) The relative mRNA level of cyclin-dependent kinase N1A (CDKN1A) after treament for 24 h. (D) The relative mRNA level of CDKN1A after treatment for 48 h.

To explore the mechanism of fangchinoline in inducing cell cycle arrest at the G0/G1 phase in K562 cells, the mRNA level of CDKN1A, which inhibits all cyclin-CDK complexes and CCND2 was investigated using quantitative real-time RT-PCR. The expression level of CCND2 markedly decreased in 1 *μ*M fangchinoline-treated cells at 24 h, whereas it significantly increased in 10 *μ*M fangchinoline-treated cells. By 48 h, the expression level of CCND2 had markedly declined in each experimental group (Fig. 3A and B). After exposure to 10 *μ*M fangchinoline for 24 and 48 h, the mRNA level of CDKN1A markedly increased compared to the control group. CDKN1A mRNA expression in other experimental groups tended to increase; however, there was no significant difference compared with the control group (Fig. 3C and D). These results indicate that cell cycle arrest at the G0/G1 phase in K562 cells may result from upregulation of CDKN1A and downregulation of CCND2.

### Fangchinoline does not induce apoptosis in K562 cells

**Figure 4:**
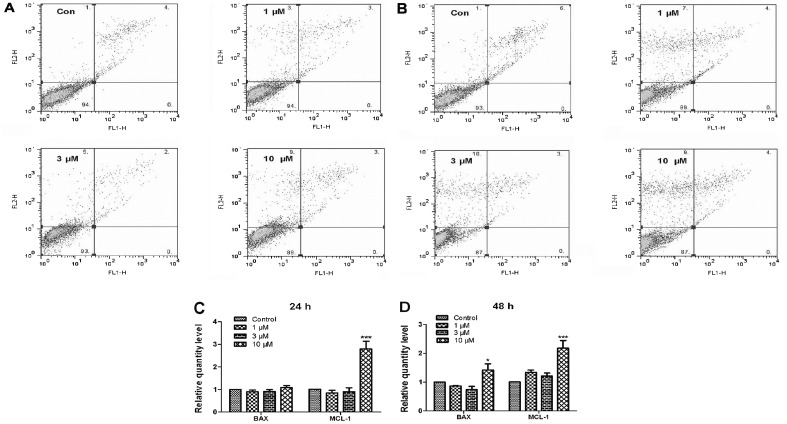
Effect of fangchinoline on cell apoptosis and apoptosis-related gene expression in K562 cells. (A) Cells were treated with and without the indicated concentrations of fangchinoline for 24 h and the apoptotic cells were determined using Annexin-V-Fluos and propidium iodide (PI) staining. (B) Cells were treated with and without the indicated concentrations of fangchinoline for 48 h and the apoptotic cells were determined using Annexin-V-Fluos and PI staining. (C) After treatment for 24 h, the mRNA levels of B cell lymphoma-2-associated X protein (BAX) and MCL-1 were determined. Data are expressed as mean ± standard deviation (SD) for three independent experiments. (D) After treatment for 48 h, the mRNA levels of BAX and MCL-1 were determined. Data are expressed as mean ± SD for three independent experiments.

To evaluate whether the anti-proliferation effect of fangchino-line is required to induce apoptosis, we detected cell apoptosis with and without fangchinoline treatment using the Annexin-V-Flous/PI dual-staining assay. As shown in Fig. 4A and B, after treatment with fangchinoline for 24 and 48 h at various concentrations, the percentages of apoptotic cells in each group were 0%, which demonstrated that fangchinoline does not induce apoptosis in K562 cells. To determine the anti-apoptotic mechanism of fangchinoline in K562 cells, we examined the mRNA levels of BCL-2 family members, including MCL-1 and BAX. Quantitative real-time RT-PCR analysis revealed that the mRNA level of MCL-1 significantly increased after treatment with 10 *μ*M fangchinoline for 24 h. The mRNA level of BAX slightly increased after treatment with 10 *μ*M fangchinoline for 48 h; however, the ratio of BAX to MCL-1 mRNA level was markedly lower than in the control group. Treatment for 24 and 48 h with 1 and 3 *μ*M fangchino-line did not induce discernible changes in the mRNA level of BAX and MCL-1 (Fig. 4C and D).

## Discussion

Previous studies have shown that a number of herbal extracts and isolated compounds possess antitumor activity. Tetrandrine is a major compound from *radix Stephaniae tetrandrae*. The potent antitumor activity of tetrandrine has been extensively reported. Fangchinoline is a derivative of tetrandrine, with structural features similar to tetrandrine. Several studies have demonstrated that fangchinoline inhibits the growth of various tumor cells and have determined the mechanisms involved in inducing G1/S phase cell cycle arrest, potentiating cancer cell apoptosis and triggering excessive autophagy instead of inducing apoptosis. However, the effects and the underlying mechanisms of fangchinoline in human CML cells remain unclear. In the present study, we observed that fangchinoline exerts a significant growth inhibition effect in K562 cells and the inhibition effects are dose- and time-dependent. Further analysis revealed that fangchinoline treatment triggers cell cycle arrest at the G0/G1 phase; however, it does not induce apoptosis of K562 cells. These effects are considered a result of the upregulation of CDKN1A and MCL-1 expression, as well as downregulation of CCND2 expression.

Cell cycle progression is precisely regulated by a series of cell cycle regulators, including cyclins, CDKS and CDK inhibitors (CDKIs). Progression through the G1 phase and transition from G1 into the S phase are regulated by cyclin D, E and their dependent kinases. D-type cyclins, including CCND1, CCND2 and CCND3, assemble with CDK4 and CDK6 to form active complexes (33), The cyclin D-CDK4/6 complexes induce the phosphorylation of the retinoblastoma (Rb) protein and the release of E2F, which triggers G1 cell cycle progression (34). Increased expression of CDKs and cyclins has been observed in the majority of cancer cells. The deregulation of the cell cycle in the G1 phase has been implicated in tumor development and proliferation (35,36). In hematopoietic cells, CCND2 and CCND3 mediate the G1-S-phase transition and, if overexpressed, allow for G1-S phase progression under conditions of growth-factor deprivation. Thus, it is likely that BCR-ABL in K562 cells provides a mitogenic signal that results in overexpression of CCND2 and facilitates the G1-S phase transition (37). In our study, we identified that fangchinoline decreases the mRNA level of CCDN2 and induces G0/G1 growth arrest in K562 cells. These partly account for K562 cell proliferation inhibition following fangchinoline treatment.

Cyclin/CDK complexes are negatively regulated by two families of CDK inhibitors. The first class of inhibitors includes the INK4a proteins. The second family of inhibitors is composed of Cip/Kip proteins, including p21, p27 and p57 (38). p21 encoded by CDKN1A is a member of the Cip/Kip family of cyclin-dependent kinase inhibitors known to be upregulated in response to DNA damage and oxidative stress and p21 plays an essential role in growth arrest following DNA damage by binding and inhibiting cyclin/CDK complexes. Expression of p21 in response to DNA damage and other cellular stress is regulated largely at the transcriptional level by p53-dependent and -independent mechanisms (39–41). p21 was originally considered a negative regulator of the cell cycle and a tumor suppressor. p21 is involved in the regulation of fundamental cellular programs, including cell proliferation, differentiation, migration, senescence and apoptosis. It not only exhibits anti-oncogenic, but also oncogenic properties. The functions of p21 depend on its intracellular localization. In the nucleus, it serves as a negative cell cycle regulator and tumor suppressor, in particular by participating in the launch of a senescence program. When p21 is localized in the cytoplasm, it acts as an oncogene by protecting cells against apoptosis (42). In addition, levels of p21 often determine the cellular response to various drugs. RKO human colorectal carcinoma cells, which express low levels of p21, normally undergo apoptosis in response to prostaglandin A2. In contrast, NIH 3T3 cells and MCF-7 cells express high levels of p21 and undergo G1 arrest in response to prostaglandin A2 (43,44). In BCR-ABL-transformed hematopoietic cells, BCR-ABL-induced expression of p21 is localized exclusively in the nucleus. In BCR-ABL-positive cells, p21 decreases cell proliferation; however, it does not change the level of spontaneous apoptosis. p21 reduces IM-and taxol-induced apoptosis in BCR-ABL-positive cells (45). In this study, we demonstrated that treatment of K562 cells with 10 *μ*M fangchinoline quickly and significantly increases the mRNA level of CDKN1A. Our results strongly suggest that p21, as a negative regulator of the cell cycle, induces cell cycle arrest at the G0/G1 phase in K562 cells treated with fangchinoline.

Chemotherapy induces apoptosis of tumor cells. In the Annexin V-Flous/PI dual-staining assay, we did not observe the occurrence of fangchinoline-induced apoptosis. BCL-2 family members are critical regulators of apoptosis (46). Proteins of this family are divided into anti- and pro-apoptotic proteins. Anti-apoptotic BCL-2 proteins, including BCL-2, BCL-XL and MCL-1, prevent the release of cytochrome *c* from mitochondria, whereas pro-apoptotic BAX and BAK participate in the formation of pores in the mitochondria through which cytochrome *c* is released (47-50). MCL-1 has been identified as a BCR/ABL-dependent survival factor in CML (51) and acts as an anti-apoptotic factor in various neoplastic cells, including several leukemia-derived cell lines (52,53). Upregulation of MCL-1 expression has been implicated in the chemoresistance of certain malignancies (54). One study demonstrated that MCL-1 inhibits BAX in the absence of MCL-1/BAX interaction and the anti-apoptotic function of MCL-1 requires the presence of BAX (55). Hence, we focused on the expression of MCL-1 and BAX in K562 cells following treatment with fangchinoline. Our data demonstrated that a high concentration (10 *μ*M) of fangchinoline increases the mRNA level of MCL-1 and BAX in K562 cells. This may be the main reason why fangchinoline is unable to induce apoptosis of K562 cells. In addition, there is accumulating evidence that CDKN1A confers a protective advantage against apoptosis, which appears to be correlated with a cytoplasmic translocation of the protein (56–58). Therefore, fangchinoline-induced upregulation of CDKN1A expression may also contribute to the survival of K562 cells. However, the inhibition of apoptotic cell death does not mean that other forms of cell death occur in K562 cells treated with fangchinoline. The MTT assay revealed that the growth inhibition rate of K562 cells reached 98% after treatment for 48 h with 10 *μ*M fangchinoline. Therefore, we hypothesize that non-apoptotic cell death occurs in K562 cells treated with 10 *μ*M fangchinoline. It is necessary to clarify the role of p21 and MCL-1 in non-apoptotic cell death.

In conclusion, fangchinoline potently increased the expression of CDKN1A and MCL-1, and decreased the expression of CCND2. Additionally, it caused cell cycle arrest at the G0/G1 phase and did not induce apoptosis, resulting in the inhibition of proliferation in K562 cells. To date, there are no reports on the adverse reaction of *radix Stephaniae tetrandrae*. Therefore, fangchinoline may be a new candidate in the therapeutic strategy of CML.
